# Psychiatry

**Published:** 1904-12-03

**Authors:** 


### PSYCHIATRY.

(*Concluded from page* 157.)

**Paranoia.**—An important discussion on this form of insanity is contributed by Dr. Percy Smith[3] in his Presidential Address to the Medico-Psychological Association The illness of the late King Ludwig II. of Bavaria, who was a victim of paranoia, and whose tragic end is within the recollection of many, is thus recorded in the certificate as to the nature of the King's insanity, signed by four physicians: "His Majesty is in a far-advanced state of insanity, suffering from that form of mental disease which is well known to alienist physicians of experience as paranoia." The socialist philosopher Rousseau was also a sufferer from paranoia, and it is believed that his death in 1778, after about 18 or 20 years of this mental affliction, was really a suicidal act. Dr. Smith, in a long review of the history of opinion on the subject of the word paranoia, concludes that the term should be limited to what is now understoood in Germany as *Verrücktheit*, or chronic mental affection marked by a slowly developing system of insane delusions tending to transformation of personality, but not to dementia. Krafft-Ebing taught that the disease originated in the "intellectual" sphere of the mind as distinguished from the "affective" sphere of feeling; that paranoia was originally a disease of the intellect. This view is rejected by Dr. Smith, who holds that the domain of organic feeling—the affective sphere of the mind—is the first to be involved in paranoia. Kraepelin, of Heidelburg, who has by his writings influenced contemporary thought in Psychiatric Medicine more than any other author, states that paranoia is not a common disease in asylums, and that it constitutes only from two to four per cent. of the admissions. Men are more often afflicted than women. The disease begins in early adult life, between the ages of 25 and 40. It develops on a defective constitution of the neuropathic or psycho-pathic type. "Peculiar traits and eccentricities may be recognised early in life, the patients being moody, dreamy, or seclusive. Some show perverted sexual instincts, or a marked aptitude for study or mental activity in special limited fields. Some have been abnormally bright; others have always been flighty, entering into many projects which they were unable to pursue successfully. Many show stigmata of degeneration." Berkley states that paranoia is more often inherited than acquired, and bears a close relationship to moral imbecility. The recognition of the disease derives its great importance in psychiatry and medical jurisprudence, not from its frequency, because it is relatively a rare form of mental alienation, but from the fact that often the affected person is a dangerous individual, frequently able to conceal his insane concepts and delusions not only from the laity but from medical men, until a deed of violence or crime suddenly perpetrated, brings to light before society the fact of the existence of a dangerous lunatic in their midst. The development of paranoia depends upon a constitutional neuropathic ground-work. This, while strikingly true of the earlier forms, is only hardly less apparent in those which come on later in life. So seldom, indeed, can the disease be traced to post-natal causes that the paranoiac may be said to be one predestined to his morbid peculiarities—one for whom there is no escape except through the channels of an especial environment. The proof of degeneracy is the unbalanced and anomalous development of the psychical functions which the paranoiac fulfils to the letter, despite his exalted position above the mental level of his half-brother the imbecile. The first steps in the evolution of the progressive and self-elaborating alienation which constitutes paranoia may often be recognised in childhood. Such children show the peculiar traits and eccentricities referred to in the foregoing paragraph. Moreover, in their school days they keep aloof from their companions of similar age; they are dreamy, irritable, and deficient in the correct understanding of ethical reasoning; they occupy themselves more with their fantasies and dream-life than with natural sport and play; they may study with apparent zest a multitude of subjects but they do not become thoroughly grounded in any one, and they are peculiarly deficient in mathematics or some other of the exact sciences. "The environment in the years of childhood and puberty," says Berkley,[4] "is all-important to such psychically defective ones. When burdened with only a moderate degree of inherited weakness they may now acquire nervous stability sufficient to enable them to pass through the early years of adult life and allow them to follow the customary occupations and pursuits of mankind. Eventually, however, they fall by the wayside, when the retrogressive period of life comes on, as it happens in every brain with inherited peculiarities; in the case of these afflicted ones this involution begins considerably earlier than in the normal man." There are two chief types of paranoia regarded from the evolutionary aspect. In the one originating in early life, the victim may be said to be a paranoiac from the cradle; in the other, the prodromes of the alienation of feeling, mood, conduct, and thought usually manifests itself about the period of puberty or adolescence. According to the age at which they arise the types are known as early and late paranoia (paranoia originäre and paranoia tardive). Both forms are essentially similar in symptomatology, the chronic progressive systematic evolution and co-ordination of delusions into an entire delusional involvement of the Ego, constituting the central feature of the disease, around which secondary symptoms of anomalies of conduct, speech, and mannerisms may be variously developed according to the circumstances of life. "We find in paranoia a distinct disease entity, separated from the psychoneuroses by its insidious development over months and years, by the retention of the reasoning process, and by the absence of pronounced dementia after the disease has progressed for many years." Kræpelin says the prognosis of the disease is very poor, as no case of genuine paranoia ever recovers.[5]

[3] Journ. of Ment. Sci., Oct. 1904. [4] A Treatise on Ment. Dis., p. 379. [5] Clin. Psych., 1902, p. 326.

